# Porous PdWM (M = Nb, Mo and Ta) Trimetallene for High C1 Selectivity in Alkaline Ethanol Oxidation Reaction

**DOI:** 10.1002/advs.202103722

**Published:** 2021-12-23

**Authors:** Yingnan Qin, Hao Huang, Wenhao Yu, Haonan Zhang, Zhenjiang Li, Zuochao Wang, Jianping Lai, Lei Wang, Shouhua Feng

**Affiliations:** ^1^ Key Laboratory of Eco‐chemical Engineering Key Laboratory of Optic‐electric Sensing and Analytical Chemistry of Life Science Taishan Scholar Advantage and Characteristic Discipline Team of Eco Chemical Process and Technology Laboratory of Inorganic Synthesis and Applied Chemistry College of Chemistry and Molecular Engineering Qingdao University of Science and Technology Qingdao 266042 P. R. China; ^2^ School of Sustainable Energy Materials and Science Jinhua Advanced Research Institute Jinhua 321000 P. R. China; ^3^ Shandong Engineering Research Center for Marine Environment Corrosion and Safety Protection College of Environment and Safety Engineering Qingdao University of Science and Technology Qingdao 266042 P. R. China; ^4^ College of Materials Science and Engineering Qingdao University of Science and Technology Qingdao 266042 China

**Keywords:** ethanol oxidation reaction, high C1 selectivity, high valence metal, metallene, Pd

## Abstract

Direct ethanol fuel cells are among the most efficient and environmentally friendly energy‐conversion devices and have been widely focused. The ethanol oxidation reaction (EOR) is a multielectron process with slow kinetics. The large amount of by‐product generated by incomplete oxidation greatly reduces the efficiency of energy conversion through the EOR. In this study, a novel type of trimetallene called porous PdWM (M = Nb, Mo and Ta) is synthesized by a facile method. The mass activity (15.6 A mg_Pd_^−1^) and C1 selectivity (55.5%) of Pd_50_W_27_Nb_23_/C trimetallene, obtained after optimizing the compositions and proportions of porous PdWM, outperform those of commercial Pt/C (1.3 A mg_Pt_^−1^, 5.9%), Pd/C (5.0 A mg_Pd_^−1^, 7.2%), and Pd_97_W_3_/C bimetallene (9.5 A mg_Pd_^−1^, 14.1%). The mechanism by which Pd_50_W_27_Nb_23_/C enhances the EOR performance is evaluated by in situ Fourier transform infrared spectroscopy and density functional theory calculations. It is found that W and Nb enhance the adsorption of CH_3_CH_2_OH and oxophilic high‐valence Nb accelerates the subsequent oxidation of CO and —CH*_x_* species. Moreover, Nb promotes the cleavage of C—C bonds and increases the C1 selectivity. Pd_60_W_28_Mo_12_/C and Pd_64_W_27_Ta_9_/C trimetallene synthesized by the same method also exhibit excellent EOR performance.

## Graphical abstract

Porous PdWM (M = Nb, Mo and Ta) trimetallenes exhibit the enhanced mass activity and selectivity for ethanol oxidation reaction in 1.0 m KOH solution. Pd_50_W_27_Nb_23_/C shows the highest mass activity of 15.6 A mg_Pd_^−1^ and C1 pathway Faradic efficiency of 55.5% at 0.77 V versus RHE. Meanwhile, there is only 30.1% mass activity decrease after 3000 cycles CV tests.

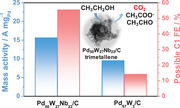

## 1 Introduction

Direct ethanol fuel cells are among the most promising energy‐conversion devices owing to their high energy density and environmental friendliness.^[^ ^1^, ^2^, ^3^, ^4^, ^5^, ^6^ ^]^ As a biomass fuel, ethanol is lowly toxic, delivers reproducible performance, and is easily stored and transported.^[^ ^7^, ^8^, ^9^, ^10^, ^11^, ^12^ ^]^ However, the ethanol oxidation reaction (EOR) at the anode of DEFCs is a multielectron process with slow kinetics, and a sizeable amount of by‐product is generated by incomplete oxidation.^[^ ^13^, ^14^, ^15^, ^16^, ^17^ ^]^ Incomplete oxidation, called the C2 pathway, transfers only four electrons and yields acetic acid/acetate (CH_3_COOH/CH_3_COO^−^). In contrast, complete oxidation (the C1 pathway) transfers 12 electrons and produces carbon dioxide/carbonate (CO_2_/CO_3_^2−^). Acetaldehyde (CH_3_CHO) is another main by‐product of two‐electron transfer.^[^ ^18^, ^19^, ^20^, ^21^ ^]^ Due to the large energy consumption of breaking the C—C bond, the C1 selectivity of Pt and Pd is very low (< 7.5%).^[^ ^22^ ^]^ Meanwhile, when strongly adsorbed intermediates (CO_ads_) occupy the active sites of catalysts, the EOR performance is significantly degraded.^[^ ^23^, ^24^, ^25^ ^]^

To avoid the above problems, the design and synthesis of EOR catalysts with high selectivity and anti‐poisoning ability is urgently demanded. In recent years, the EOR performances of Pd‐based catalysts have been widely evaluated in alkaline solution. Owing to their higher oxophilicity than Pt, these catalysts outperform Pt in EOR catalysis.^[^ ^26^, ^27^, ^28^, ^29^ ^]^ Oxophilic groups (such as Bi, Ni, Cu, Sn, CeO_2_, and WO*_x_*) adsorb OH_ads_, promoting the further oxidation of CO and ‐—CH*_x_* species.^[^ ^30^, ^31^, ^32^, ^33^, ^34^ ^]^ Equally important is weakening the adhesion energy between the toxic species and the catalyst surface. For this purpose, the electron structures of Pd must be regulated. The electron structure of Pd is usually optimized by regulating its composition (for example, with metal or nonmetal doping)^[^ ^35^, ^36^, ^37^ ^]^ and morphology (such as metallene, core/shell and defect).^[^ ^38^, ^39^, ^40^ ^]^ Currently, Pd‐based metallenes are regarded as star materials owing to their special architectural feature.^[^ ^41^, ^42^ ^]^ First, the ultrathin 2D structure increases the electrochemical active area (ECSA) and improves the atomic availability of Pd. Second, the curved structure induces a distinct strain effect that can optimize the electron structure of Pd and enhance the intrinsic activity of as‐prepared catalysts. Owing to the quantum size effect and strain effect originating from the ultrathin curved geometry, the ORR (oxygen reduction reaction) mass activity of PdMo bimetallene is 78 times higher than that of commercial Pt/C in alkaline solution.^[^ ^43^ ^]^ Defect‐rich porous PdW metallene has achieved enhanced ORR performance in alkaline solution. In this structure, the oxygen binding ability on Pd was optimized by the strain effect and tunable electronic structure derived from the highly curved sub‐nanometer nanosheet.^[^ ^44^ ^]^ PdIr bimetallene achieves higher HER (hydrogen evolution reaction) and FAOR (formic acid oxidation reaction) performances than commercial Pt/C and Pd/C. As revealed in density functional theory (DFT) calculations, the concave‐convex structure produces a special strain effect and optimizes the electron environment for the HER and FAOR.^[^ ^45^ ^]^ Although bimetallenes that improve the ORR, FAOR and HER have been investigated in depth, trimetallenes have rarely been studied.

Herein, we present a facile synthesis of porous PdWM (M = Nb, Mo and Ta) trimetallene with high C1 selectivity for the alkaline EOR. The mass activity of porous Pd_50_W_27_Nb_23_/C trimetallene is 12.0, 3.3, and 1.6 times higher than those of Pt/C, Pd/C and Pd_97_W_3_/C bimetalline, respectively. The anti‐CO poisoning ability of Pd_50_W_27_Nb_23_/C also exceeds those of other as‐prepared catalysts, because the oxophilic high‐valence Nb accelerates the subsequent oxidation of CO and —CH*_x_* species. In a gas chromatography analysis of the EOR products, the Pd_50_W_27_Nb_23_/C exhibited much higher C1 selectivity than Pt/C, Pd/C, and Pd_97_W_3_/C. In situ Fourier transform infrared (FTIR) and DFT calculations indicated that W and Nb enhance the adsorption of CH_3_CH_2_OH, and Nb, thus promoting C—C bond cleavage and increasing the C1 selectivity. It is worth noting that Pd_60_W_28_Mo_12_/C and Pd_64_W_27_Ta_9_/C, which also exhibit excellent EOR mass activity and C1 selectivity, can be synthesized by the same method.

## 2 Results and Discussions

**Figure 1:**
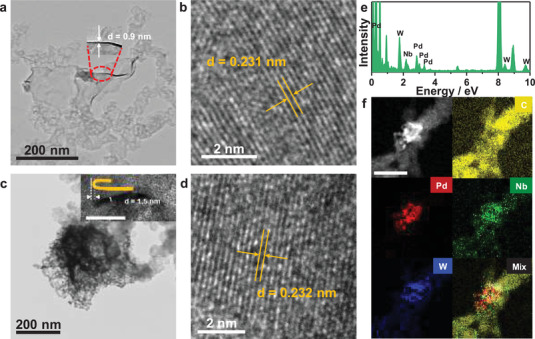
Physical characterization of as‐prepared catalysts. a,b) TEM and HRTEM images of Pd_97_W_3_/C. c,d) TEM and HRTEM images, e) EDS spectrum, and f) EDS‐mapping image of Pd_50_W_27_Nb_23_/C. The inset in (c) is the vertical part of as‐prepared Pd_50_W_27_Nb_23_/C, the scale bar is 20 nm.

The porous Pd_97_W_3_ bimetallenes were synthesized from Pd(acac)_2_ and W(CO)_6_ precursors by a one‐pot wet‐chemical method. The CO prolysized from W(CO)_6_ functioned as a capping reagent in the formation of ultrathin metallene. The porous structure was formed by etching with acetic acid. The morphology of the as‐prepared catalysts was characterized by transmission electron microscopy (TEM) and scanning electron microscopy (SEM). The ultrathin Pd_97_W_3_/C was around 0.9 nm thick (**Figure** **1**a; Figure S1a, b, Supporting Information) and quite wrinkled. In the high‐resolution TEM (HRTEM) image (Figure 1b), the lattice distance of Pd_97_W_3_ was determined as 0.231 nm, corresponding to the (111) facet of as‐prepared Pd_97_W_3_ bimetallene. The three main XRD peaks located at 39.5°, 45.2° and 67.1° were indexed to the (111), (200) and (220) facets, respectively, of the face‐centered cubic (fcc) structure. The peak positions were obviously negatively shifted from those of the standard PDF card (JCPDS # 46–1043) of Pd, reflecting the lattice distortion and strain effect caused by the highly curved structure. Adapting the mature synthesis method of Pd_97_W_3_ bimetallene, we successfully synthesized PdWNb trimetallenes from NbCl_5_ precursor. The as‐prepared Pd_50_W_27_Nb_23_ was highly porous and stacked into structures with a thickness of around 1.5 nm, conferring a largely increased specific surface area and fast mass transfer efficiency (Figure 1c; Figure S2a,b, Supporting Information). The HRTEM image (Figure 1d) reveals a lattice distance of 0.231 nm in Pd_50_W_27_Nb_23_. As evidenced in energy disperse spectroscopy (EDS) mapping (Figure 1e,f), the Pd, W, and Nb elements were uniformly dispersed through PdWNb trimetallene at a composition ratio of 50.1: 27.7: 22.2. The main XRD peaks in Figure S2c (Supporting Information) are slightly negatively shifted from those of Pd, indicating small expansion of the lattice. The morphologies of the as‐prepared Pd_44_W_37_Nb_19_ (Figure S3a,b, Supporting Information) and Pd_31_W_22_Nb_47_ (Figure S3d–f, Supporting Information) are similar to those of Pd_97_W_3_ and Pd_50_W_27_Nb_23_. The XRD patterns (Figure S3c, e, Supporting Information) indicate a fcc structure of Pd_44_W_37_Nb_19_ and Pd_31_W_22_Nb_47_. The atomic ratio of each element was analyzed using inductively coupled plasma atomic emission spectroscopy (ICP‐AES) (Table S1, Supporting Information). To deeply understand the valences of the elements in the as‐prepared catalysts, we analyzed the XPS spectra of Pd_97_W_3_/C, Pd_50_W_27_Nb_23_/C, Pd_44_W_37_Nb_19_/C, and Pd_31_W_22_Nb_47_/C. The peak positions of Pd_50_W_27_Nb_23_/C, Pd_44_W_37_Nb_19_/C and Pd_31_W_22_Nb_47_/C are shifted by −0.22, −0.25, and −0.30 eV from those of Pd_97_W_3_/C, respectively (Figure S4a, Supporting Information). As confirmed in the XPS spectra of W 4f and Nb 3d (Figure S4b, c, Supporting Information), W and Nb are completely oxidized, so the negative shift of the XPS peak of Pd is likely caused by electron transfer from the higher‐valence metals (Nb, W) to Pd.

**Figure 2:**
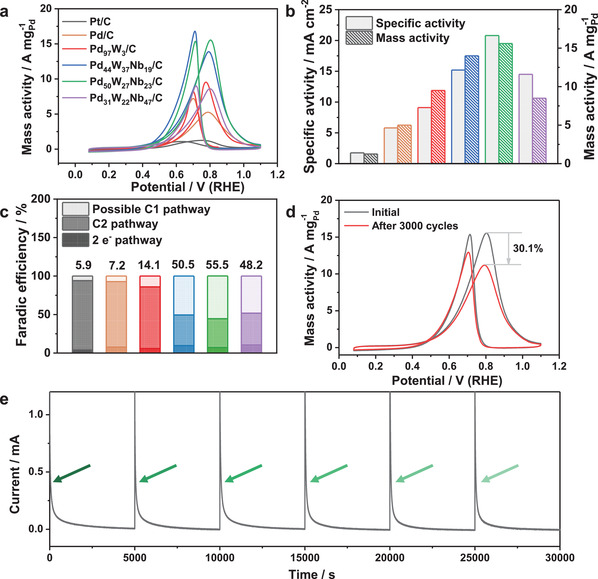
Ethanol oxidation reaction performance of as‐prepared PdWNb trimetallene. a) CV curves in N_2_‐saturated 1.0 m KOH contained 1.0 m ethanol with a scan rate of 50 mV s^−1^. b) Histogram of specific activity and mass activity. c) Faradic efficiency of as‐prepared PdWNb/C in different reaction pathway at 0.77 V versus RHE. d) CVs of Pd_50_W_27_Nb_23_/C before and after 3000 cycles. e) *i*–*t* curves of Pd_50_W_27_Nb_23_/C, and the arrows indicate when the electrolyte is refreshed.

The EOR performances of as‐prepared PdWNb/C trimetallenes were tested on a CHI 660E electrochemical workstation (Chenhua, Shanghai) using a traditional three‐electrode system. Prior to electrochemical tests, the as‐prepared catalysts were dispersed in a mixture of water, isopropanol and 5 wt% Nafion solution (v: v: v = 1: 1: 0.01), forming a uniform catalyst ink at a concentration of 1 mg mL^−1^. All catalysts were surface‐cleaned by activation with cyclic voltammetry (CVs) in N_2_‐saturated 1.0 m KOH. The Pd_50_W_27_Nb_23_/C catalyst exhibits a lower onset potential and higher intensity ratio of forward peak to backward peak (*I*_f_/*I*_b_) than the other catalysts, indicating its superior antipoisioning performance (**Figure** **2**a). The EOR specific activity and mass activity of Pd_50_W_27_Nb_23_/C are 20.8 mA cm^−2^ and 15.6 A mg_Pd_^−1^, respectively (Figure 2b), which is 11.6 and 12.0 times higher than commercial Pt/C (1.8 mA cm^−2^, 1.3 A mg_Pd_^−1^), 3.5 and 3.3 times higher than commercial Pd/C (5.9 mA cm^−2^, 5 A mg_Pd_^−1^), 2.3 and 1.6 times higher than Pd_97_W_3_/C bimetallene (9.1 mA cm^−2^ and 9.5 A mg_Pd_^−1^). Meanwhile, the Pd_44_W_37_Nb_19_/C (15.2 mA cm^−2^, 14.0 A mg_Pd_^−1^) and Pd_31_W_22_Nb_47_/C (14.5 mA cm^−2^, 8.5 A mg_Pd_^−1^) trimetallenes outperformed Pt/C and Pd_97_W_3_/C for the EOR. Furthermore, the CO_ads_ species is an important intermediate product of the EOR that can adhere to the catalyst surface and poison the active sites, thus decreasing the electrochemical performance. The CO resistances of the catalysts were thus evaluated in a CO‐stripping test (Figure S5a, Supporting Information). The CO oxidation peak of Pd_50_W_27_Nb_23_/C appears at a lower potential than the peaks of the other catalysts, indicating that Pd_50_W_27_Nb_23_/C better resists CO than the other catalysts. The oxophilic high‐valence Nb can accelerate the subsequent oxidation of CO and —CH*_x_* species and eliminate the influence of CO poisoning. The ECSAs of the as‐prepared catalysts were calculated from the integral areas of the CO oxidation peaks (Figure S5b, Supporting Information). Pd_97_W_3_/C shows a much larger ECSA (104 m^2^ g^−1^) than commercial Pt/C (71.5 m^2^ g^−1^) and Pd/C (85.2 m^2^ g^−1^). The ECSA gradually decreases with increasing Nb addition (100.3, 75.2, and 58.6 m^2^ g^−1^ in Pd_44_W_37_Nb_19_/C, Pd_50_W_27_Nb_23_/C, and Pd_31_W_22_Nb_47_/C, respectively; Table S2, Supporting Information).

To further study the C1/C2 selectivity of the as‐prepared catalysts, we detected the possible 2e^−^ and C2 products (acetaldehyde and acetic acid) by GC. We first obtained the current–time (*i*–*t*) curves of Pd_50_W_27_Nb_23_/C at different applied potentials and calculated the Faradic efficiencies (FE) of the 2e^−^ and C2 products from the standard curves (Figure S6, Supporting Information). As shown in Figure S7 (Supporting Information), the FEs of acetaldehyde and acetic acid decreased as the applied potential increased from 0.57 V to 0.77 V versus RHE but increased when the applied potential reached 0.87 V (vs RHE). Therefore, when investigating the C1/C2 selectivity of the as‐prepared catalysts, we set the applied potential to 0.77 V (vs RHE). Assuming a total C1 and C2 pathway FE of 100%, the possible FE of Pd_50_W_27_Nb_23_/C in C1 reached 55.5% at 0.77 V (vs RHE). By the same method, the C1 FEs of commercial Pt/C, commercial Pd/C, Pd_97_W_3_/C, Pd_44_W_37_Nb_19_/C, and Pd_31_W_22_Nb_47_/C were calculated as 5.9%, 7.2%, 14.1%, 50.5%, and 54.2%, respectively (Figure 2c) (Table S3, Supporting Information). The results indicate that the Nb element promotes C—C bond breaking and enhances the C1 selectivity of the Pd_50_W_27_Nb_23_/C catalyst.

**Figure 3:**
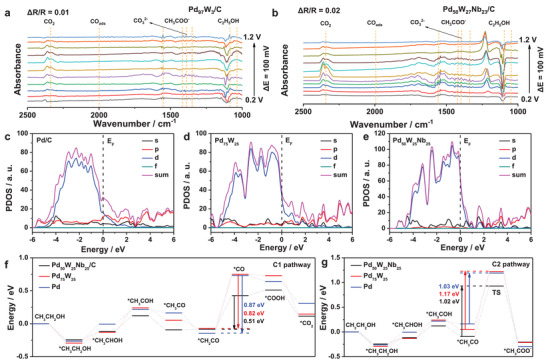
In situ FTIR spectra of ethanol oxidation reaction on the a) Pd_97_W_3_/C and b) Pd_50_W_27_Nb_23_/C. Electronic properties of Pd, Pd_75_W_25_, and Pd_50_W_25_Nb_25_ slab. The PDOS plots of c) Pd, d) Pd_75_W_25_, and e) Pd_50_W_25_Nb_25_ slab. Free energy diagram of e) C1 and g) C2 pathways in Pd, Pd_75_W_25_, and Pd_50_W_25_Nb_25_ slabs.

To clarify the reaction mechanism, the intermediate products during the EOR process were detected by in situ FTIR. The characteristic bands at 1045 and 1087 cm^−1^ in the spectra of Pd_97_W_3_/C and Pd_50_W_27_Nb_23_/C are assigned to C—O stretching in ethanol, and the downward trend of these peaks indicates the consumption of ethanol (**Figure** **3**a,b). The higher peak intensity of Pd_50_W_27_Nb_23_/C than of Pd_97_W_3_/C indicates that ethanol adsorption was enhanced by introducing Nb. The peak at 1348 cm^−1^ belongs to ‐CH_3_ bending vibrations of acetate, while the peaks at 1414 and 1550 cm^−1^ are assigned to symmetric and asymmetric stretching bands of O—C—O in acetate ions (CH_3_COO^−^), respectively. Meanwhile, the characteristic band of CO_3_^2–^ at 1390 cm^−1^ is more intense in the spectrum of Pd_50_W_27_Nb_23_/C than in the spectrum of Pd_97_W_3_/C, indicating the higher C1 selectivity of Pd_50_W_27_Nb_23_/C than of Pd_97_W_3_/C. The peak at 2000 cm^−1^ assigned to CO adsorption (CO_ads_) gradually diminishes with increasing applied potential, reflecting the faster CO oxidation and desorption from the surface of catalysts. The peak at 2340 cm^−1^ is assigned to asymmetric stretching of O—C—O in CO_2_. Evidently, Pd_50_W_27_Nb_23_/C produces much more CO_2_ than Pd_97_W_3_/C at the same potential, indicating that the Nb additive accelerates the cleavage of C—C bands and increases the C1 selectivity of the EOR.

To thoroughly understand the enhanced EOR performance of PdWNb, the possible reaction mechanism was elucidated in DFT calculations. The role of each element in the EOR reaction was evaluated on Pd, Pd_75_W_25_ and Pd_50_W_25_Nb_25_ slabs built in the model (Figure S8a, Supporting Information). The electronic properties were determined from the projected partial density of states (PDOS) of Pd in each slab (Figure 3c–e). First, the wide energy band of the Pd‐4d orbital can enhance the adsorption of CH_3_CH_2_OH and CH_3_CO on the catalyst surface, providing favorable activation conditions for the EOR. Second, after incorporating the W and Nb elements, the energy difference between the d‐band center of Pd‐4d and the Fermi level gradually decreases from 1.02 to 0.78 eV and then to 0.20 eV. As the d‐band center more closely approaches the Fermi level, the electron transfer from d orbitals to the adsorbed CH_3_CH_2_OH occurs more rapidly, which can enhance the adsorption of reactant. Therefore, it can be concluded that W and Nb promote the adsorption of CH_3_CH_2_OH on the catalyst surface, facilitating the subsequent EOR and improving the EOR performance.

To understand the excellent C1 selectivity of the as‐prepared PdWNb, the free energy changes were determined along the C1 and C2 paths of the reaction. Figure S8b (Supporting Information) shows the EOR reaction path of PdWNb. As is well known, the greatest energy impediment along the C1 path is C—C bond cleavage and subsequent oxidation. During the C—C bond cleavage step, when *CH_3_CO evolves into *CO, the energy barrier is smaller over PdWNb than over the other catalysts (Figure 3f) (0.51 eV vs 0.87 eV for Pd and 0.82 eV for PdW), promoting C—C bond cleavage and improving the C1 selectivity. On the contrary, the C—C bond of the *CH_3_OH species cannot be broken along the C2 pathway and further oxidation to CH_3_COO^−^ is possible. As evidenced in Figure 3g, the oxidation of *CH_3_OH to *CH_3_COO^−^ must overcome a huge reaction energy barrier (1.03 eV for Pd, 1.17 eV for PdW, and 1.02 eV for PdWNb), so the reaction is driven toward the C1 pathway. After introducing Nb, the energy input of C—C bond breakage is greatly reduced and the C1 selectivity is increased.

Stability is another major indicator of catalyst performance. After 3000 CV cycles, the mass activity of Pd_50_W_27_Nb_23_/C was reduced by 30.1% (Figure 2d), much lower than in commercial Pt/C (69.5%), Pd/C (63.5%), Pd_97_W_3_/C (51.0%), Pd_44_W_37_Nb_19_/C (45.1%), and Pd_31_W_22_Nb_47_/C (41.8%) (Figure S9, Supporting Information). Moreover, Pd_50_W_27_Nb_23_/C maintained its original morphology and chemical status after 3000 cycles (Figures S10 and S11, Supporting Information), indicating the excellent structural and electronic stability of this catalyst. Meanwhile, after 5000 *i*–*t* tests, the mass activity decrease of the Pd_50_W_27_Nb_23_/C was only 15.4%, the lowest among the tested catalysts (Figure S12, Supporting Information). After six continuous *i–t* tests, the original catalytic activity of Pd_50_W_27_Nb_23_/C was recovered after refreshing the electrolyte (Figure 2e). The rapid current decay during the initial stage is caused by lowering of the concentration gradient and poisoning by intermediate species (CO, C_2_H_5_OH, and CH_3_CHO).^[^ ^46^, ^47^, ^48^ ^]^

**Figure 4:**
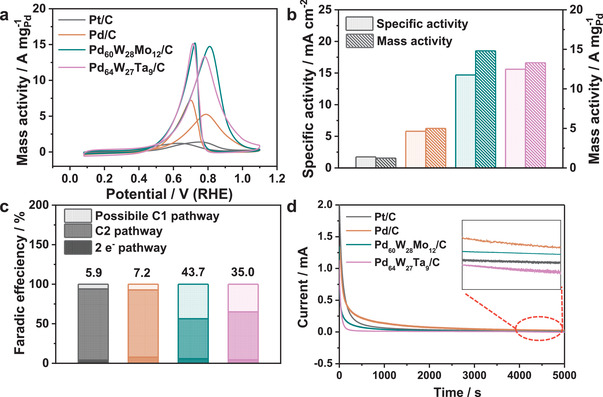
Ethanol oxidation reaction performance of as‐prepared PdWM trimetallene. a) CV curves in N_2_‐saturated 1.0 m KOH contained 1.0 m ethanol with a scan rate of 50 mV s^−1^. b) Histogram of specific activity and mass activity. c) Faradic efficiency of as‐prepared PdWM/C in different reaction pathway at 0.77 V versus RHE. d) *i*–*t* curve of PdWM/C at 0.77 V versus RHE for 5000 s.

The synthesis strategy of PdWM trimetallene is generalizable to other high‐valence metals such as Mo and Ta. When NbCl_5_ was replaced with MoCl_5_ and TaCl_5_, the same synthesis yielded Pd_60_W_28_Mo_12_/C and Pd_64_W_27_Ta_9_/C trimetallenes, respectively, which (like Pd_50_W_27_Nb_23_/C) possessed porous and wrinkled structures (see Figure S13a,b,d,e, Supporting Information). The slight negative shift in the XRD spectra of Pd_60_W_28_Mo_12_/C and Pd_64_W_27_Ta_9_/C is attributable to the alloy effect (Figure S13c,f, Supporting Information). In the XPS spectra, the Pd positions of Pd_60_W_28_Mo_12_/C and Pd_64_W_27_Ta_9_/C are negatively shifted by 0.22 and 0.18 eV, respectively, from that of Pd_97_W_3_/C, indicating that Pd is an electron acceptor (Figure S14a, Supporting Information). Meanwhile, W, Mo and Ta mainly exist in the oxidation state (Figure S14b–d, Supporting Information). The EOR performances of Pd_60_W_28_Mo_12_/C and Pd_64_W_27_Ta_9_/C were studied in 1.0 M KOH. The mass and specific activities were 14.8 A mg_Pd_^−1^and 14.7 mA cm^−2^, respectively, in Pd_60_W_28_Mo_12_/C and 13.3 A mg_Pd_^−1^ and 15.6 mA cm^−2^, respectively, in Pd_64_W_27_Ta_9_/C (**Figure** **4**a,b). These performances approach those of Pd_50_W_27_Nb_23_/C. The high CO resistances of Pd_60_W_28_Mo_12_/C and Pd_64_W_27_Ta_9_/C were confirmed in CO stripping tests (Figure S15a, Supporting Information). Both catalysts exhibited higher ECSA than commercial Pt/C (Figure S15b, Supporting Information). The possible C1 selectivities of Pd_60_W_28_Mo_12_/C and Pd_64_W_27_Ta_9_/C were 43.3% and 35.0% respectively (Figure 4c). After 3000 CV cycles, the mass activities of Pd_60_W_28_Mo_12_/C and Pd_64_W_27_Ta_9_/C were decreased by 31.9% and 59.7%, respectively (Figure S16, Supporting Information). After 5000 *i–t* tests, the mass‐activity reductions of Pd_60_W_28_Mo_12_/C and Pd_64_W_27_Ta_9_/C were only 13.6% and 39.8%, respectively (Figure 4d and Figure S17, Supporting Information). Overall, the Pd_60_W_28_Mo_12_/C and Pd_64_W_27_Ta_9_/C catalysts prepared by the same strategy as Pd_50_W_27_Nb_23_/C presented similarly high EOR activities and stabilities, demonstrating the universality of the one‐pot wet chemical synthesis strategy.

## 3 Conclusions

In summary, porous PdWM (M = Nb, Mo and Ta) trimetallenes exhibit excellent EOR performance and high C1 selectivity. The porous, wrinkled structure enlarges the ECSA, accelerates the electron and mass transfer, and modifies the electron structure of the as‐prepared catalysts. The mass activity of porous Pd_50_W_27_Nb_23_/C trimetallene (15.6 A mg_Pd_^−1^) was 12.0, 3.3, and 1.6 times that of commercial Pt/C (1.3 A mg_Pt_^−1^), Pd/C (5.0 A mg_Pd_^−1^) and bimettaline Pd_97_W_3_/C (9.5 A mg_Pd_^−1^), respectively, and the possible C1 selectivity was 55.5%, much higher than those of Pt/C (5.9%), Pd/C (7.2%), and Pd_97_W_3_/C (14.1%). In DFT calculations, the W and Nb additives were found to enhance the adsorption of CH_3_CH_2_OH and accelerate the subsequent oxidation. Nb also promotes C‐C bond cleavage and increases the C1 selectivity. The same method synthesized Pd_60_W_28_Mo_12_/C and Pd_64_W_27_Ta_9_/C with similarly excellent EOR mass activity (14.8 and 13.3 A mg_Pd_^−1^, respectively) and C1 selectivity (43.7% and 35.0%, respectively). This study introduced the synthesis and application of trimetallenes and demonstrated that introducing high‐valence metals can promote C‐C bond cleavage. Furthermore, this work will facilitate the study of other electrocatalyst reactions.

## Conflict of Interest

The authors declare no conflict of interest.

## Supporting information

Supporting Information

## Data Availability

Research data are not shared.
