# Supplementary material for: Porous PdWM (M = Nb, Mo and Ta) Trimetallene for High C1 Selectivity in Alkaline Ethanol Oxidation Reaction
Source: Adv Sci (Weinh). 2021 Dec 23;9(5):2103722. doi: 10.1002/advs.202103722 (PMC8844492; doi:10.1002/advs.202103722)
Supplement: Supplementary file 1 — Supporting Information [file ADVS-9-2103722-s001.pdf]

ADVANCED SCIENCE
Open Access

Supporting Information

for *Adv. Sci.*, DOI: 10.1002/advs.202103722

Porous PdWM (M = Nb, Mo and Ta) Trimetallene for High C1 Selectivity in Alkaline Ethanol Oxidation Reaction

Yingnan Qin[#], Hao Huang[#], Wenhao Yu, Haonan Zhang, Zhenjiang Li, Zuochao Wang, Jianping Lai[*], Lei Wang[*], Shouhua Feng

Copyright WILEY-VCH Verlag GmbH & Co. KGaA, 69469 Weinheim, Germany, 2021.

Supporting Information

Porous PdWM (M = Nb, Mo and Ta) Trimetallene for High C1 Selectivity in Alkaline Ethanol Oxidation Reaction

Yingnan Qin#, Hao Huang#, Wenhao Yu, Haonan Zhang, Zhenjiang Li, Zuochao Wang, Jianping Lai, Lei Wang*, Shouhua Feng*

Experimental Section

Chemicals. Palladium (II) acetylacetonate ($Pd(acac)_2$, 99%) was purchased from Sigma-Aldrich. Tungsten hexacarbonyl ($W(CO)_6$, 97%) and commercial Pt/C (20 wt%) purchased from Alfa Aesar. Molybdenum(V) chloride ($MoCl_5$, 99.5%) and niobium(V) chloride ($NbCl_5$,99.9%) were obtained from MACKLIN. Tantalic chloride ($TaCl_5$, 95%), potassium hydroxide (KOH, 85%) and acetic acid (CH_3COOH, 99.5%) were obtained from Aladdin. N, N-Dimethylformamide (DMF, 99.5%) and ethanol (CH_3CH_2OH, 99.8%) was obtained from Xilong Scientific Co., Ltd. All the chemicals were used without further purification. All the solutions were prepared by high purity water (18.2 MΩ cm).

Pretreatment of Carbon. In order to remove the metallic impurity on carbon and promote functional modification of carbon, 500 mg of Ketjen Black was dispersed in 30 mL of concentrated nitric acid (65%), and reflux at 80℃ for 12 h. The product was collected by suction filtration with water to neutral, and dried at 60℃ in vacuum oven for overnight.

Preparation of $Pd_{97}W_3$/C bimetallene. 10 mg of $Pd(acac)_2$ and 20 mg $W(CO)_6$ and 8 mL of DMF were dispersed in a 15 mL pressure bottle by sonicating for 30 min, and then 2 mL of acetic acid was added into the above clarified solution. Afterwards, the bottle was put into an oil bath at 50℃ for 1 h, and 2 mL DMF contained 10 mg of treated carbon was added into the above solution and kept at 50℃ for another 1 h. The precipitate was collected by centrifugation with ethanol for three times and dried at 60℃ for overnight.

Preparation of PdWM/C (M = Mo, Ta and Nb) trimetallene. The synthesis of PdWM/C was similar with $Pd_{97}W_3$/C, except the addition of 3 mg $MoCl_5$, 4 mg $TaCl_5$ and $NbCl_5$. For $Pd_{44}W_{37}Nb_{19}$/C and $Pd_{31}W_{22}Nb_{47}$/C, the dosage of $NbCl_5$ was changed to 2 mg and 10 mg respectively.

Characterization. The morphologies of as-prepared catalysts were conducted by transmission electron microscope (TEM, HITACHI HT7800) at 100 kV and scanning electron microscope (SEM, HITACHI regulus8100) at 15 kV. The high-resolution transmission microscopy (HRTEM) images and energy dispersive X-ray spectroscopy (EDS) were taken by JEOL JEM-F200. Powder X-ray diffraction (XRD)

spectra were recorded on an X'Pert-Pro X-ray powder diffractometer equipped with a Cu radiation source (λ = 0.15406 nm). The chemical valence of each element was collected by X-ray photoelectron spectra (XPS) on SSI SProbe XPS Spectrometer. The composition of as-prepared samples was collected by the inductively coupled plasma-atomic emission spectroscopy (ICP-AES, Agilent 8800). The detection of acetic acid and acetaldehyde were conducted by gas chromatography (GC, SHIMADZU GC2014C). The intermediate products were detected by *in-situ* FTIR (Thermo iS50 FT-IR).

Electrochemical EOR measurements. Before the electrochemical tests, the as-prepared catalysts were dispersed in a mixture of water, isopropanol and 5 wt% Nafion solution (v: v: v = 1: 1: 0.01) with a concentration of 1 mg mL^{-1}. All the electrochemical tests were conducted by CHI660E electrochemical workstation (Chenhua, Shanghai) with a traditional three-electrode system. The catalysts modified glass carbon electrode was used as working electrode, a Pt foil was used as counter electrode, and a saturated calomel electrode (SCE) was used as reference electrode. The potential was calibrated by the Nernst equation that $E_{RHE} = E_{SCE} + 0.242 + 0.0592$ pH. Before each test, GCE was polished by Al_2O_3 powder to get a smooth surface. The modified working electrodes were activated by cyclic voltammetry between 0.08-1.2 V (*vs.* RHE) at 500 mV s^{-1} in N_2-saturated 1.0 M KOH for 100 cycles to get a clean catalytic surface. EOR tests were measures in N_2-saturated 1.0 M KOH contained 1.0 M ethanol between 0.08-1.1 V (*vs.* RHE) at 50 mV s^{-1}. For EOR stability tests, 3000 cycles CV tests were conducted at 0.08-1.1 V (*vs.* RHE) at 500 mV s^{-1}. The chronoamperometry tests were measured at 0.77 V for 5000 s. The CO stripping tests were carried out by i-t test in CO-saturated 1.0 M KOH at 0.1 V (*vs.* RHE) for 900 s, and then performed 2 laps of CV test.

Calculation of C1 selectivity: The yield of possible EOR product can be conducted by GC and Faraday formula. Firstly, a series of acetic acid and acetaldehyde standard solutions has been made with a stepped concentration (0.1-5 ppm). The standard curve can be drawn with the integral area of GC peak and the concentration of standard solution. Secondly, a long time i-t test has been conducted to collect the product to be measure. The concentration of as-produced acetic acid and acetaldehyde can be calculated by the standard curve. Finally, based on the Faraday formula, the Faradic efficiency of as-produced acetic acid and acetaldehyde can be calculated as follow:

$$FE=(N\times n\times 96485)/Q\times 100\%$$

N is the moles of products, n is the number of electron transfer, Q is the total amount of consumed charge during i-t test. The total FE of EOR was assumed as 100%, the possible C1 selectivity is the residue of C2 pathway.

Electrochemical *in-situ* FTIR test: The intermediate products during the EOR process were detected by *in-situ* FTIR through Thermo iS50 FT-IR with a liquid-nitrogen-cooled MCT-A detector. The in-situ FTIR curves were collected by the method of internal reflection. Firstly, the catalysts modified silicon crystal plated with gold was used as working electrode, Ag/AgCl and Pt wire were worked as reference electrode and counter electrode respectively. All the tests were conducted in N_2 saturated 0.1 M KOH with 0.5 M ethanol. The applied potential was stepped positively from 0.1 V to 1.2 V (*vs.* RHE) with an interval of 100

mV. Secondly, the results of *in-situ* FTIR were reported as relative change in absorbance: $\Delta R/R = (R(E_S)-R(E_R))/R(E_R)$. The $R(E_S)$ and $R(E_R)$ are the spectra collected at the applied potential and reference potential (0.1 V *vs.* RHE). The upward bands represent the formation of products, the downward bands represent the consumption of reactants.

Calculation Setup. DFT calculations of Pd, $Pd_{75}W_{25}$ and $Pd_{50}W_{25}Nb_{25}$ slabs were computed by using a generalized gradient approximation (GGA) of exchange-correlation functional in the Perdew, Burke, and Ernzerhof (PBE). A plane-wave energy cut off of 400 eV was used together with norm-conserving pseudopotentials, and the Brillouin zone was sampled with a 2 × 2 × 1 Monkhorst–Pack grid. The structure was fully optimized until the force on each atom is less than 10^{-3} eV/Å. To avoid periodic interaction, a vacuum layer of 30 Å was incorporated into the slabs. The free energy (G) was computed from $G = E + ZPE - T\Delta S$, where E was the total energy, ZPE was the zero-point energy, the entropy (ΔS) of each adsorbed state were yielded from DFT calculation, whereas the thermodynamic corrections for gas molecules were from standard tables.

Figures

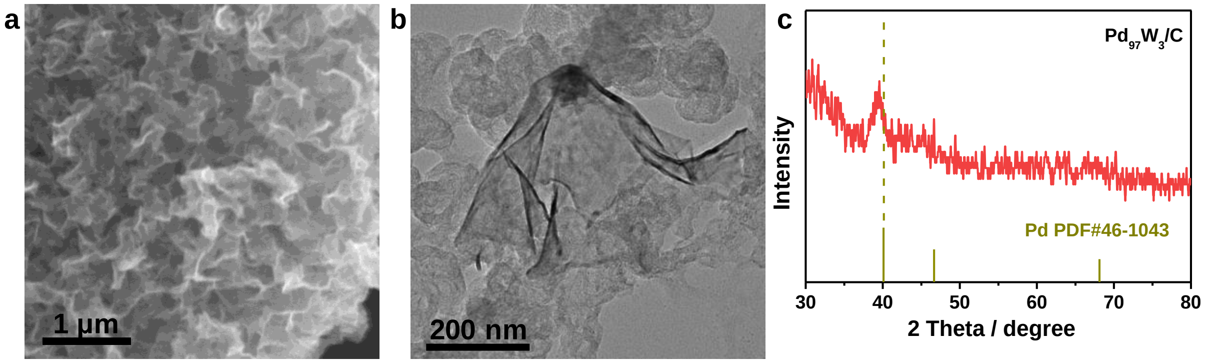

Figure S1. a) SEM, b) TEM images and c) XRD spectrum of $Pd_{97}W_3/C$ bimetallene.

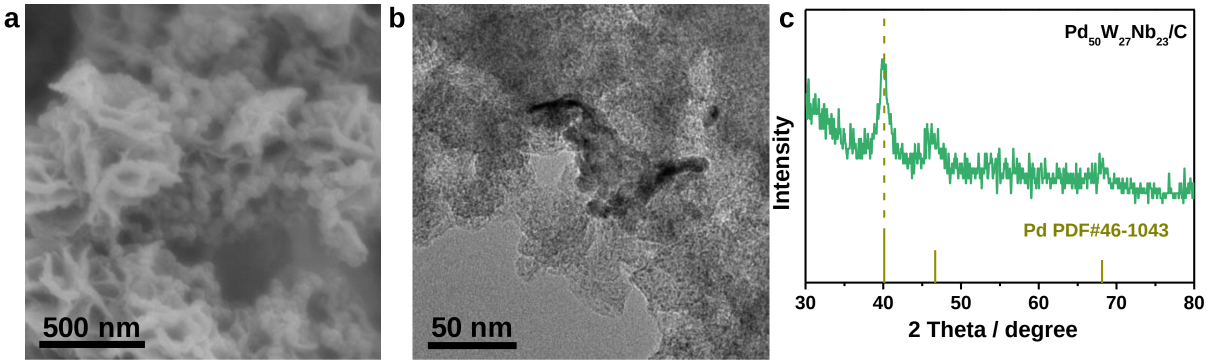

Figure S2. a) SEM, b) TEM images and c) XRD spectrum of $Pd_{50}W_{27}Nb_{23}/C$ trimetallene.

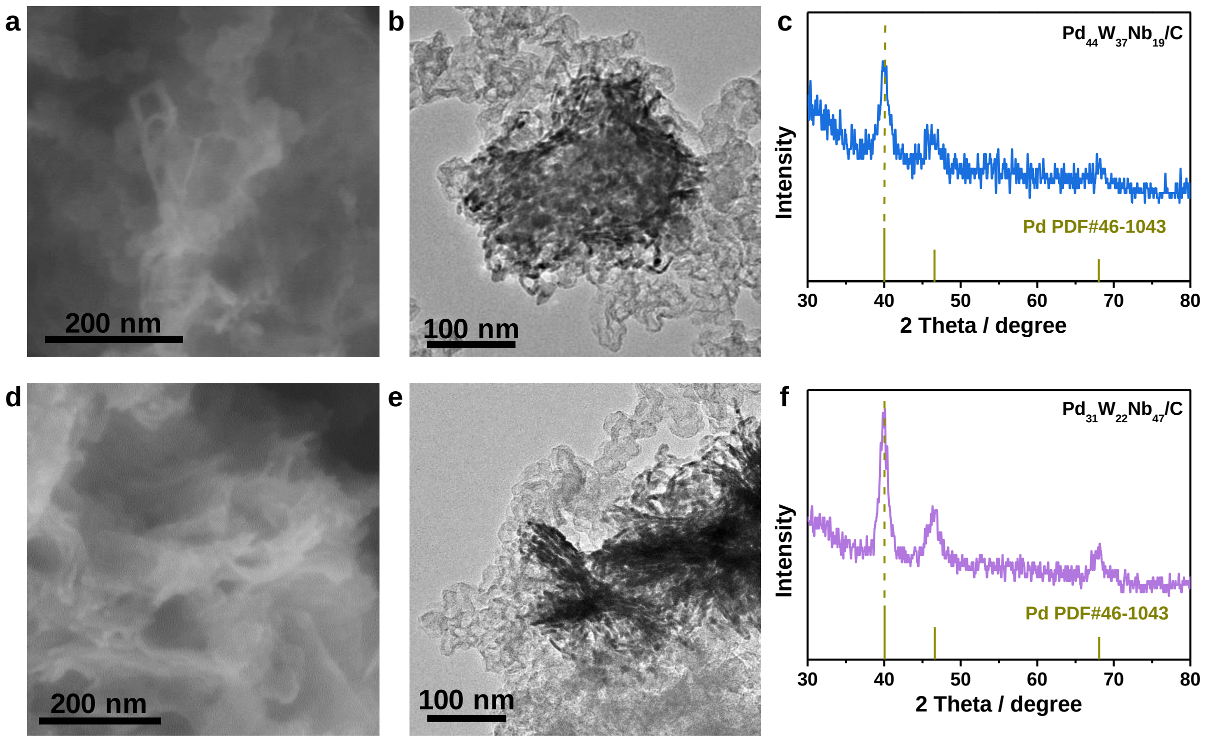

Figure S3. a, d) SEM, b, e) TEM images and c, f) XRD spectrum of $Pd_{44}W_{37}Nb_{19}/C$ and $Pd_{31}W_{22}Nb_{47}/C$ trimetallene.

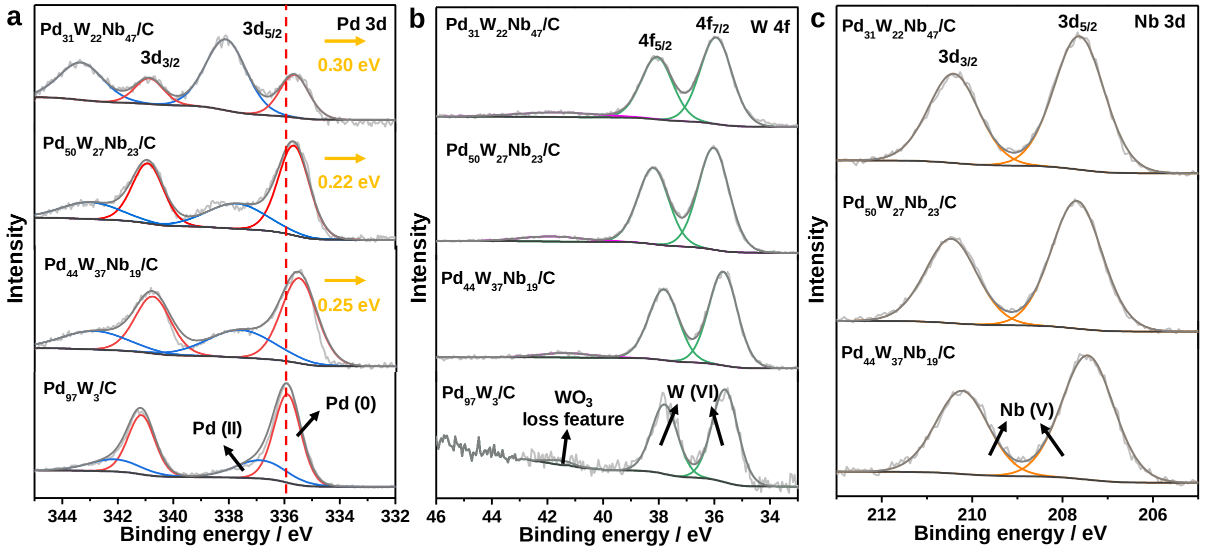

Figure S4. XPS spectrums of as-prepared catalysts. a) Pd 3d; b) W 4f; c) Nb 3d.

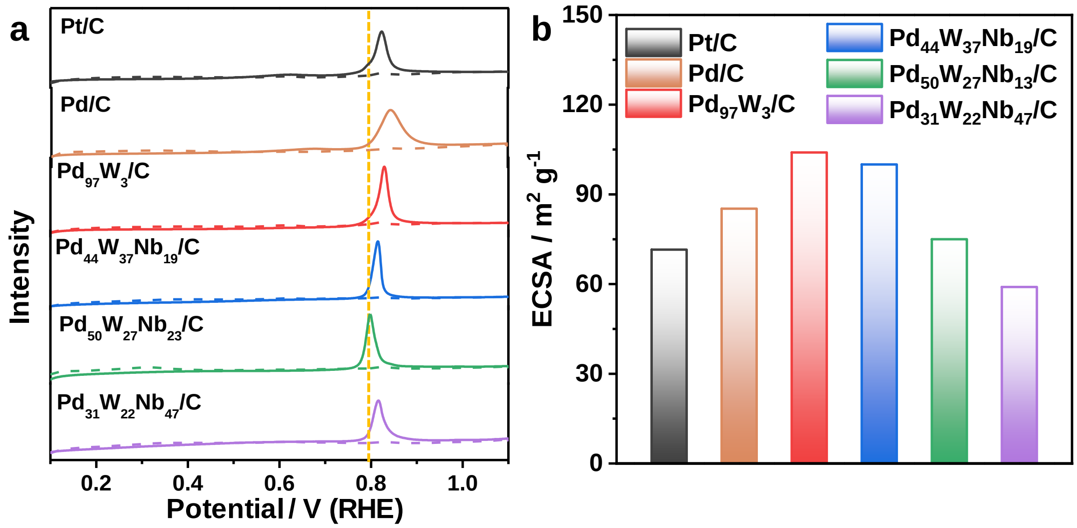

Figure S5. a) CO stripping curves of as-prepared catalysts; b) histogram of ECSAs.

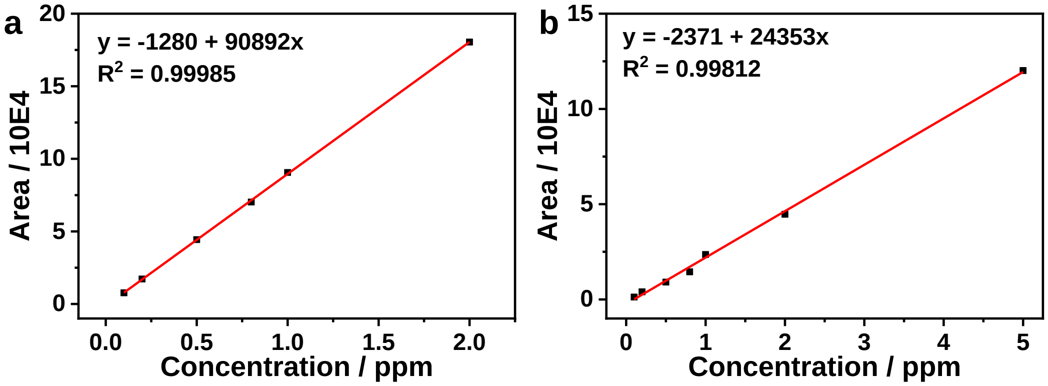

Figure S6. The standard curves of a) acetate and b) acetaldehyde in GC.

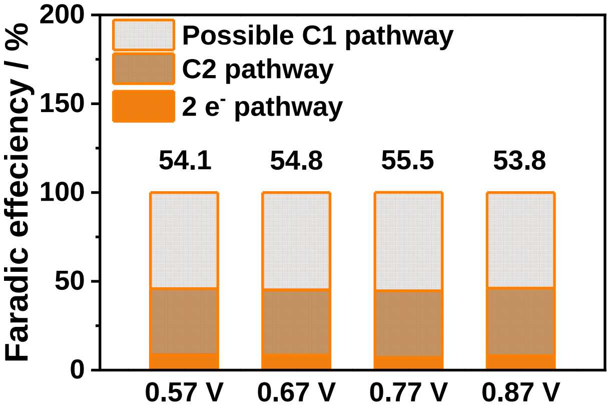

Figure S7. EOR faradic efficiency of $Pd_{50}W_{27}Nb_{23}/C$ at different applied potential.

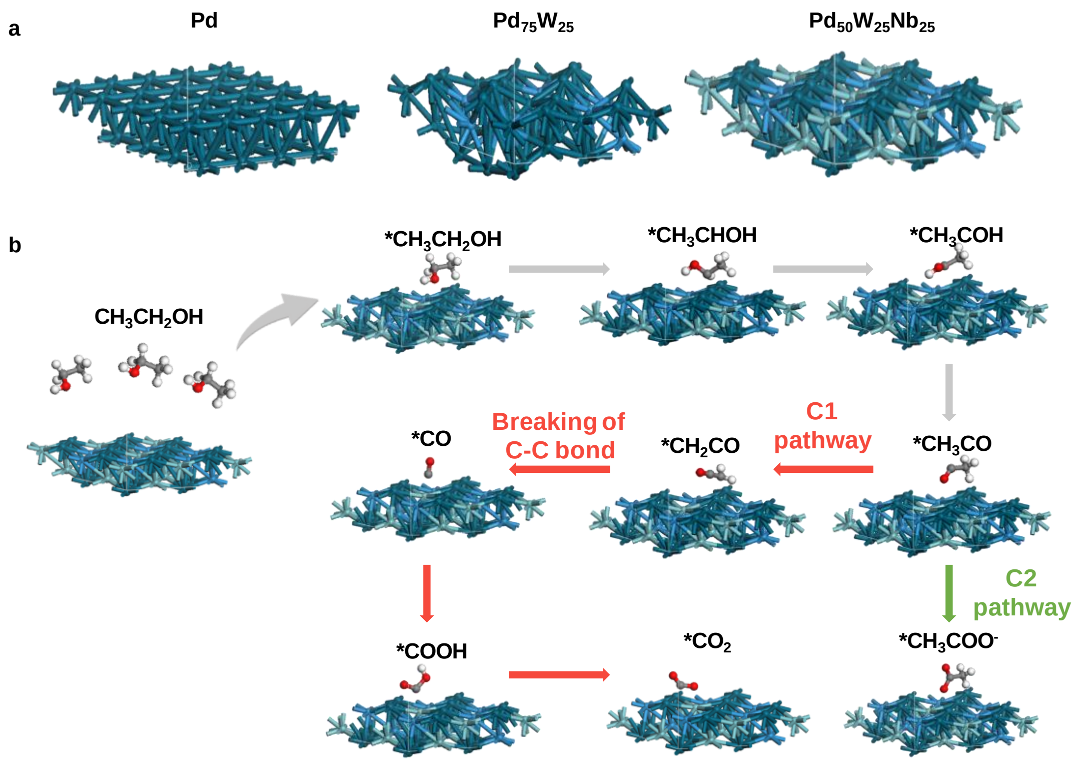

Figure S8. a) The real-spatial contour plots for each slab. b) Illustration of EOR pathway on $Pd_{50}W_{25}Nb_{25}$ slab.

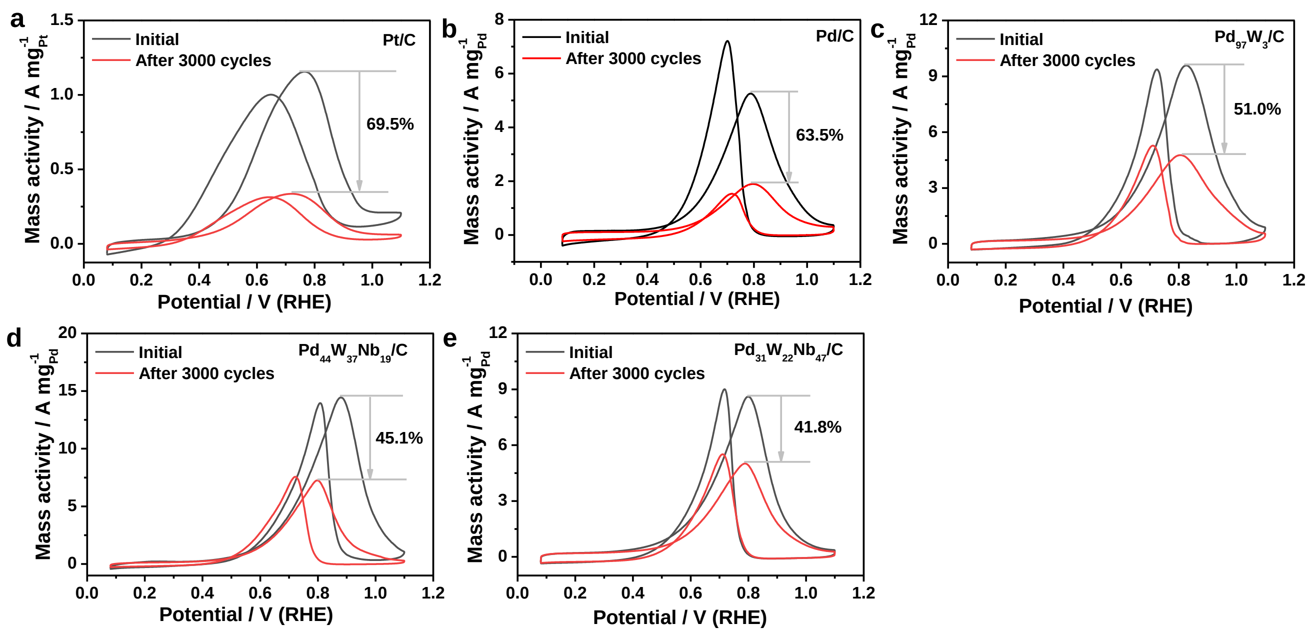

Figure S9. CV curves of as-prepared catalysts before and after 3000 cycles test. a) commercial Pt/C; b) commercial Pd/C; c) $Pd_{97}W_3$/C; d) $Pd_{44}W_{37}Nb_{18}$/C; e) $Pd_{31}W_{22}Nb_{47}$/C.

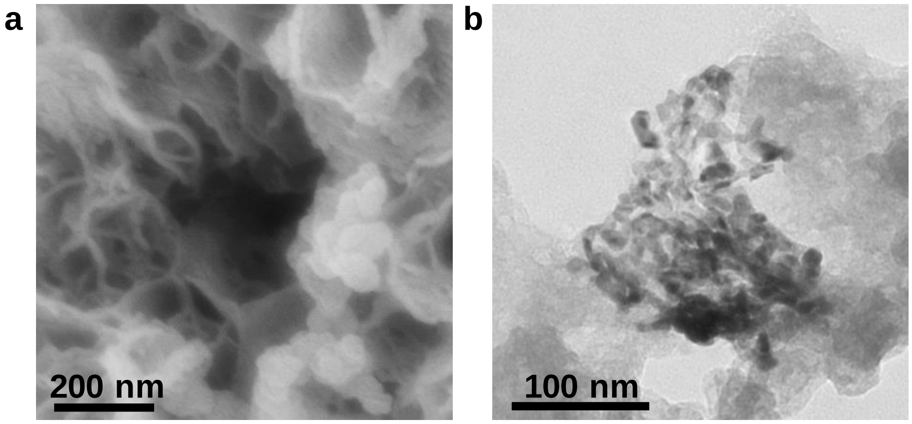

Figure S10. SEM and TEM images of $Pd_{50}W_{27}Nb_{23}/C$ after 3000 cycles CV test.

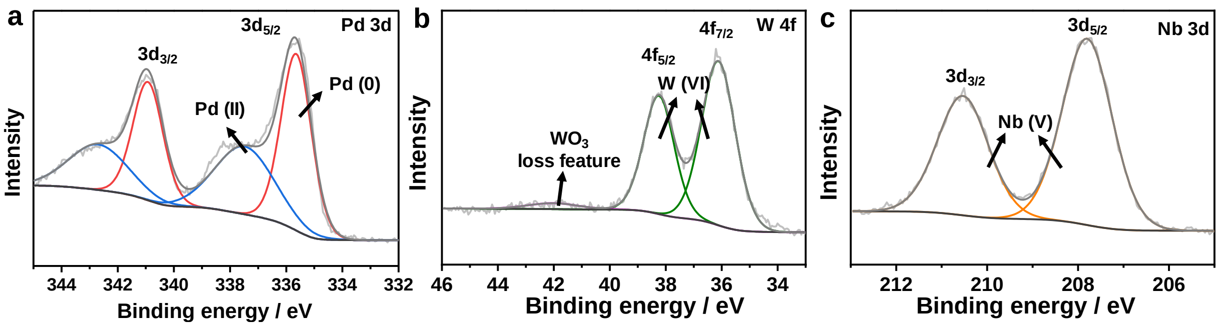

Figure S11. XPS spectrums of $Pd_{50}W_{27}Nb_{23}/C$ after a long time durability test. a) Pd 3d; b) W 4f; c) Nb 3d.

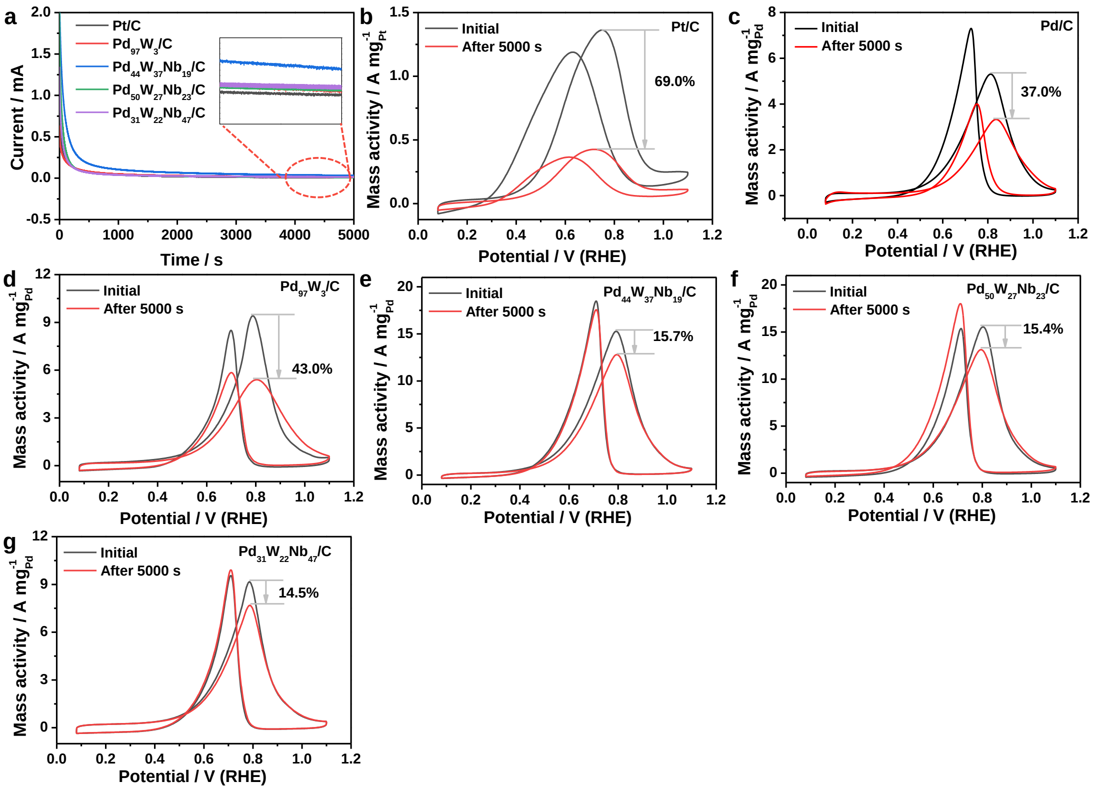

Figure S12. a) EOR i-t curves of as-prepared catalysts at 0.77 V (*vs.* RHE) for 5000 s; CV curves of as-prepared catalysts before and after 5000 s i-t tests. b) commercial Pt/C; c) commercial Pd/C; d) $Pd_{97}W_3/C$; e) $Pd_{44}W_{37}Nb_{19}/C$; f) $Pd_{50}W_{27}Nb_{23}/C$; g) $Pd_{31}W_{22}Nb_{47}/C$.

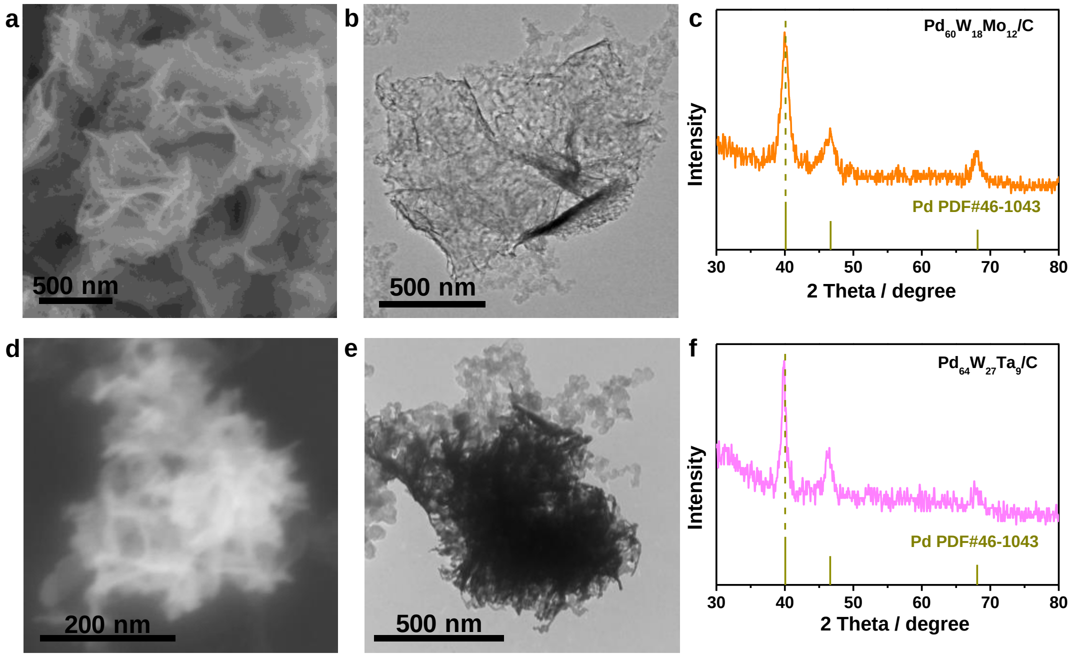

Figure S13. a, d) SEM, b, e) TEM images and c, f) XRD spectrums of $Pd_{60}W_{28}Mo_{12}/C$ and $Pd_{64}W_{27}Ta_9/C$ trimetallene.

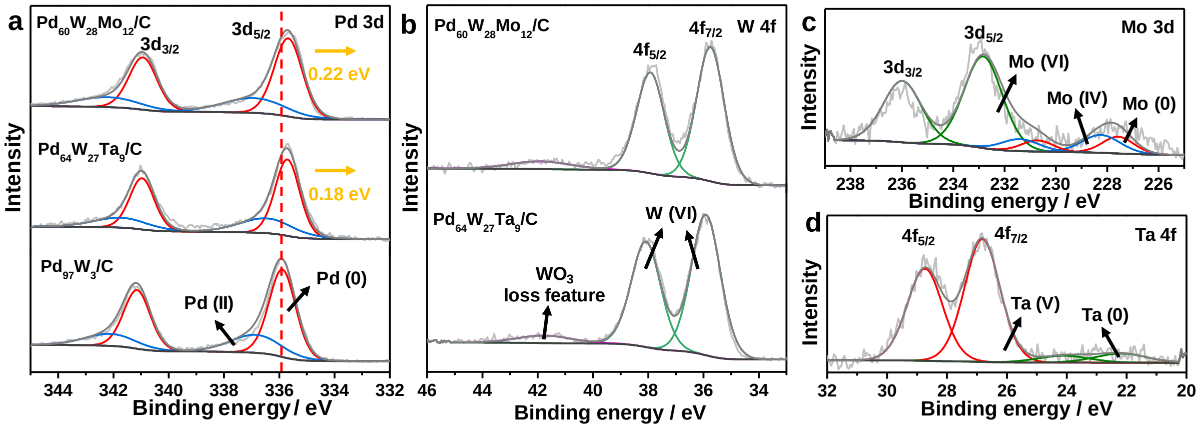

Figure S14. XPS spectrums of as-prepared catalysts. a) Pd 3d; b) W 4f; c) Mo 3d; d) Ta 4f.

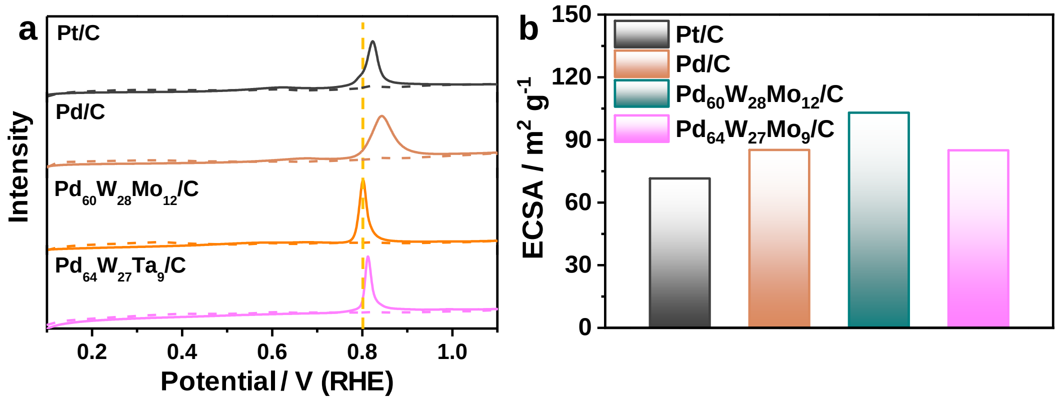

Figure S15. a) CO stripping curves of as-prepared catalysts; b) histogram of ECSAs.

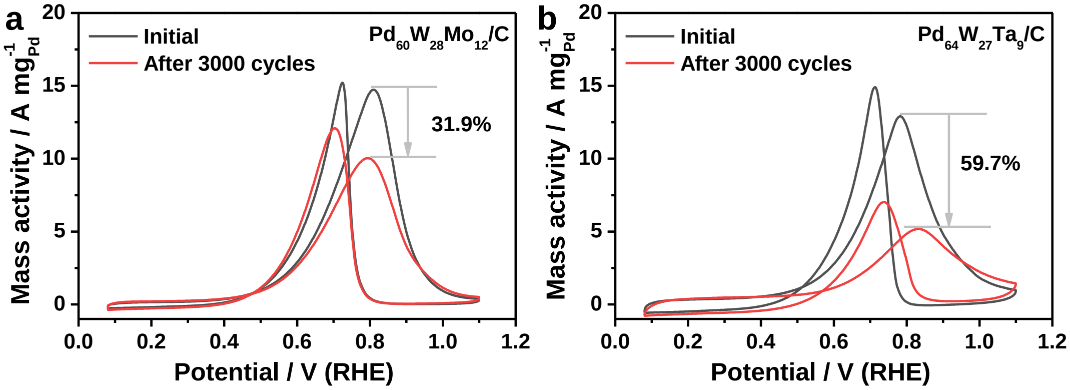

Figure S16. CV curves of as-prepared catalysts before and after 3000 cycles tests. a) $Pd_{60}W_{28}Mo_{12}/C$; b) $Pd_{64}W_{27}Ta_{9}/C$.

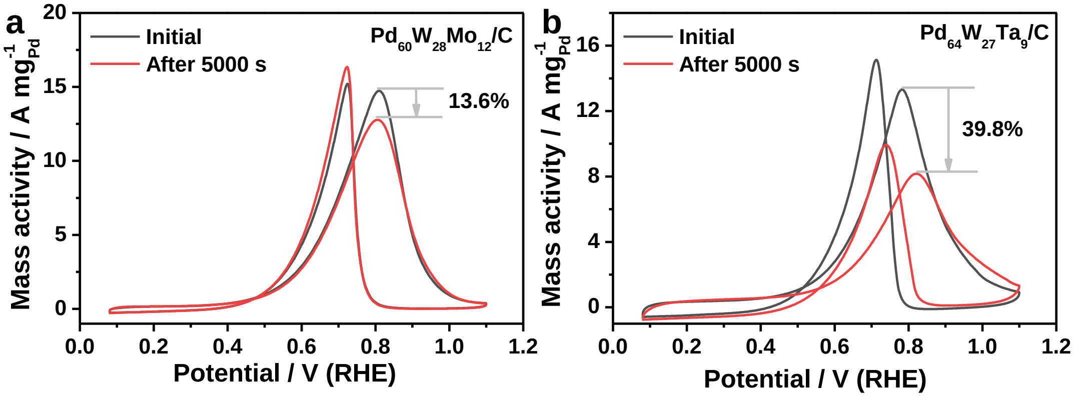

Figure S17. CV curves of as-prepared catalysts before and after 5000 s i-t tests. a) $Pd_{60}W_{28}Mo_{12}/C$; b) $Pd_{64}W_{27}Ta_{9}/C$.

Tables

Table S1. Atomic ratio of as-prepared catalysts conducted by ICP-AES

Samples	Atomic ratio / %		
	Pd	W	M
$Pd_{97}W_3/C$	96.7	3.3	—
$Pd_{44}W_{37}Nb_{19}/C$	44.1	36.8	19.1
$Pd_{50}W_{27}Nb_{23}/C$	50.2	27.3	22.5
$Pd_{31}W_{22}Nb_{47}/C$	30.8	21.7	47.5
$Pd_{60}W_{28}Mo_{12}/C$	59.8	28.4	11.8
$Pd_{46}W_{27}Ta_9/C$	64.3	27.1	8.6

Table S2. ECSAs of as-prepared catalysts

Samples	ECSA / m^2 g^{-1}
Pt/C	71.5
Pd/C	85.2
$Pd_{97}W_3/C$	104.2
$Pd_{44}W_{37}Nb_{19}/C$	100.3
$Pd_{50}W_{27}Nb_{23}/C$	75.2
$Pd_{31}W_{22}Nb_{47}/C$	58.6
$Pd_{60}W_{28}Mo_{12}/C$	102.8
$Pd_{46}W_{27}Ta_9/C$	85.1

Table S3. EOR Faradaic efficiency of as-prepared catalysts

Samples	Faradaic efficiency / %		
	**Possible C1 pathway**	**C2 pathway**	**$2e^-$ pathway**
Pt/C	5.9	89.9	4.2
Pd/C	7.2	85.0	7.8
$Pd_{97}W_3/C$	14.1	80.1	5.8
$Pd_{44}W_{37}Nb_{19}/C$	50.5	39.9	9.6
$Pd_{50}W_{27}Nb_{23}/C$	55.5	37.5	7.1
$Pd_{31}W_{22}Nb_{47}/C$	48.2	41.1	10.4
$Pd_{60}W_{28}Mo_{12}/C$	43.7	50.5	5.8
$Pd_{46}W_{27}Ta_9/C$	35.0	60.6	4.4

Table S4. Comparison performance of $Pd_{50}W_{27}Nb_{23}/C$ trimetallene and Pd-based electrocatalysts for alkaline EOR.

Catalysts	Electrolyte	Mass activity / A mg^{-1}	FE of C1 pathway / %	Reference
$Pd_{50}W_{27}Nb_{23}/C$ trimetallene	1.0 M KOH + 1.0 M ethanol	15.6	55.5	This work
Pd-Au HNS	1.0 M KOH + 1.0 M ethanol	8.0	33.2	[1]
$Ag@Pd_2P_{0.2}$	1.0 M KOH + 1.0 M ethanol	7.24	19	[2]
edge-richest 9.0 nm-$Pd_{61}Pt_{22}Cu_{17}$ nanorings	1.0 M KOH + 1.0 M ethanol	12.42	7	[3]
polycrystalline Pd disk	1.0 M NaOH + 1.0 M ethanol	NA	2.5	[4]

$Pd/Ni(OH)_2/rGO$	1.0 M KOH + 1.0 M ethanol	1.5	26	[5]
CoP/RGO-Pd	1.0 M KOH + 1.0 M ethanol	4.60	27.6	[6]
$Bi(OH)_3/PdBi$	1.0 M NaOH + 1.0 M ethanol	5.30	NA	[7]
L-PdW NAs	1.0 M KOH + 1.0 M ethanol	6.79	NA	[8]
c-Pd-Ni-P@ a-Pd-Ni-P	1.0 M KOH + 1.0 M ethanol	3.05	NA	[9]
$Pd_{87}Cu_{13}$ PNM	1.0 M KOH + 1.0 M ethanol	3.22	NA	[10]
PdBi-0.5 NCs/C	1.0 M KOH + 1.0 M ethanol	3.49	NA	[11]
HD-PdZn NCs	1.0 M KOH + 1.0 M ethanol	3.45	NA	[12]
4H/fcc Au@Pd NRs	1.0 M KOH + 1.0 M ethanol	2.92	NA	[13]
Pd-Ni-P NPs	1.0 M NaOH + 1.0 M ethanol	4.95	NA	[14]

NA: Not available.

References

[1] F. Lv, W. Zhang, M. Sun, F. Lin, T. Wu, P. Zhou, W. Yang, P. Gao, B. Huang, S. Guo, *Adv. Energy Mater.*, **2021**, *11*, 2100187.

[2] X. Yang, Z. Liang, S. Chen, M. Ma, Q. Wang, X. Tong, Q. Zhang, J. Ye, L. Gu, N. Yang, *Small*, **2020**, *16*, 2004727.

[3] W. Wang, X. Zhang, Y. Zhang, X. Chen, J. Ye, J. Chen, Z. Lyu, X. Chen, Q. Kuang, S. Xie, Z. Xie, *Nano Lett.*, **2020**, *20*, 5458.

[4] Z. Zhou, Q. Wang, J. Lin, N. Tian, S. Sun, *Electrochim. Acta*, **2010**, *55*, 7995.

[5] W. Huang, X. Y. Ma, H. Wang, R. Feng, J. Zhou, P. Duchesne, P. Zhang, F. Chen, N. Han, F. Zhao, J. Zhou, W. B. Cai, Y. Li, *Adv. Mater.*, **2017**, *29*, 1703057.

[6] M. Wang, R. Ding, Y. Xiao, H. Wang, L. Wang, C. M. Chen, Y. Mu, G. Wu, B. Lv, *ACS Appl. Mater. Interfaces*, **2020**, *12*, 28903.

[7] X. Yuan, Y. Zhang, M. Cao, T. Zhou, X. Jiang, J. Chen, F. Lyu, Y. Xu, J. Luo, Q. Zhang, Y. Yin, *Nano Lett.*, **2019**, *19*, 4752.

[8] F. Wang, S. Wang, D. Wu, H. Huang, W. Yuan, L. Y. Zhang, *Appl. Surf. Sci.*, **2021**, *537*, 147860.

[9] P. Yin, M. Zhou, J. Chen, C. Tan, G. Liu, Q. Ma, Q. Yun, X. Zhang, H. Cheng, Q. Lu, B. Chen, Y. Chen, Z. Zhang, J. Huang, D. Hu, J. Wang, Q. Liu, Z. Luo, Z. Liu, Y. Ge, X. J. Wu, X. Du, H. Zhang, *Adv. Mater.*, **2020**, *32*, 2000482.
[10] Y. Teng, K. Guo, D. Fan, H. Guo, M. Han, D. Xu, J. Bao, *Chem. Eur. J.*, **2021**, *27*, 1-9.
[11] X. Li, H. You, C. Wang, D. Liu, R. Yu, S. Guo, Y. Wang, Y. Du, *J Colloid. Interface Sci.*, **2021**, *591*, 203.
[12] S. Huang, S. Lu, H. Hu, F. Xu, H. Li, F. Duan, H. Zhu, H. Gu, M. Du, *Chem. Eng. J.*, **2021**, *420*, 130503.
[13] Y. Chen, Z. Fan, Z. Luo, X. Liu, Z. Lai, B. Li, Y. Zong, L. Gu, H. Zhang, *Adv. Mater.*, **2017**, *29*, 1701331.
[14] L. Chen, L. Lu, H. Zhu, Y. Chen, Y. Huang, Y. Li, L. Wang, *Nat. Commun.*, **2017**, *8*, 14136.
